# Germline mutations in penetrant cancer predisposition genes are rare in men with prostate cancer selecting active surveillance

**DOI:** 10.1002/cam4.4778

**Published:** 2022-04-25

**Authors:** Lauren Brady, Lisa F. Newcomb, Kehao Zhu, Yingye Zheng, Hilary Boyer, Navonil De Sarkar, Jesse K. McKenney, James D. Brooks, Peter R. Carroll, Atreya Dash, William J. Ellis, Christopher P. Filson, Martin E. Gleave, Michael A. Liss, Frances Martin, Todd M. Morgan, Ian M. Thompson, Andrew A. Wagner, Colin C. Pritchard, Daniel W. Lin, Peter S. Nelson

**Affiliations:** ^1^ Division of Human Biology Fred Hutchinson Cancer Center Seattle Washington USA; ^2^ Division of Public Health Sciences Fred Hutchinson Cancer Center Seattle Washington USA; ^3^ Department of Urology University of Washington Seattle Washington USA; ^4^ Robert J. Tomsich Pathology and Laboratory Medicine Institute Cleveland Clinic Cleveland Ohio USA; ^5^ Department of Urology Stanford University Stanford California USA; ^6^ Department of Urology University of California San Francisco California USA; ^7^ VA Puget Sound Health Care Systems Seattle WA USA; ^8^ Department of Urology Emory University School of Medicine Atlanta Georgia USA; ^9^ Winship Cancer Institute Emory Healthcare Atlanta Georgia USA; ^10^ Department of Urologic Sciences University of British Columbia Vancouver British Columbia Canada; ^11^ Department of Urology University of Texas Health Sciences Center San Antonio Texas USA; ^12^ Department of Urology Eastern Virginia Medical School Virginia Beach Virginia USA; ^13^ Department of Urology University of Michigan Ann Arbor Michigan USA; ^14^ CHRISTUS Medical Center Hospital San Antonio Texas USA; ^15^ Division of Urology Beth Israel Deaconess Medical Center Boston Massachusetts USA; ^16^ Department of Laboratory Medicine and Pathology University of Washington Seattle WA USA

**Keywords:** active surveillance, adverse pathology, germline mutations, prostate cancer

## Abstract

**Background:**

Pathogenic germline mutations in several rare penetrant cancer predisposition genes are associated with an increased risk of aggressive prostate cancer (PC). Our objectives were to determine the prevalence of pathogenic germline mutations in men with low‐risk PC on active surveillance, and assess whether pathogenic germline mutations associate with grade reclassification or adverse pathology, recurrence, or metastases, in men treated after initial surveillance.

**Methods:**

Men prospectively enrolled in the Canary Prostate Active Surveillance Study (PASS) were retrospectively sampled for the study. Germline DNA was sequenced utilizing a hereditary cancer gene panel. Mutations were classified according to the American College of Clinical Genetics and Genomics' guidelines. The association of pathogenic germline mutations with grade reclassification and adverse characteristics was evaluated by weighted Cox proportional hazards modeling and conditional logistic regression, respectively.

**Results:**

Overall, 29 of 437 (6.6%) study participants harbored a pathogenic germline mutation of which 19 occurred in a gene involved in DNA repair (4.3%). Eight participants (1.8%) had pathogenic germline mutations in three genes associated with aggressive PC: *ATM*, *BRCA1*, and *BRCA2*. The presence of pathogenic germline mutations in DNA repair genes did not associate with adverse characteristics (univariate analysis HR = 0.87, 95% CI: 0.36–2.06, *p* = 0.7). The carrier rates of pathogenic germline mutations in *ATM*, *BRCA1*, and *BRCA2*did not differ in men with or without grade reclassification (1.9% vs. 1.8%).

**Conclusion:**

The frequency of pathogenic germline mutations in penetrant cancer predisposition genes is extremely low in men with PC undergoing active surveillance and pathogenic germline mutations had no apparent association with grade reclassification or adverse characteristics.

## Graphical abstract

Low‐risk prostate cancer is generally managed using active surveillance with deferred aggressive treatment. Pathogenic germline mutations in several rare penetrant cancer predisposition genes such as BRCA2 are associated with an increased risk of aggressive prostate cancer and could influence treatment choice. We ascertained the prevalence of pathogenic mutations in men with low risk prostate cancer enrolled on an active surveillance study. The frequency of pathogenic germline mutations in penetrant cancer predisposition genes was extremely low in men on active surveillance and no association with grade reclassification or adverse characteristics during surveillance or unfavorable outcomes after treatment was identified.

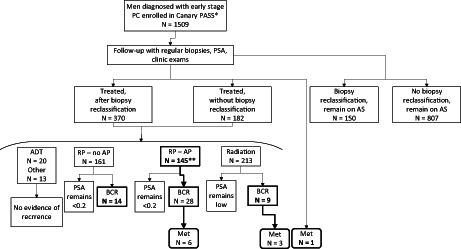

## 1 INTRODUCTION

An increasing number of men initially diagnosed with low‐risk prostate cancer (PC) are now managed using active surveillance (AS)^1^ whereby patients undergo periodic reassessments of laboratory, biopsy, and imaging studies designed to evaluate changes in tumor characteristics that may warrant curative intervention. While AS comprises a standard of care pathway adopted by national guidelines, the variable natural history of cancers initially considered low‐risk in addition to the limitations of prostate biopsy that may under‐sample the presence of aggressive PC, remain concerns that influence treatment choice.^2^, ^3^ To address this issue, biomarkers capable of providing more individualized assessments of clinical outcomes have been actively sought.^3^, ^4^, ^5^

In addition to features measured directly from tumors, host characteristics, such as pathogenic germline mutations, can also serve as biomarkers that associate with cancer outcomes in a number of malignancies.^6^ In PC, pathogenic germline mutations in highly penetrant cancer predisposition genes have been associated with poor disease‐specific outcomes. For example, compared to men with localized PC, men with metastatic PC have higher rates of pathogenic germline mutations, particularly in DNA repair genes (DRGs), indicating that heritable alterations in DNA repair pathways contribute to aggressive behavior.^7^ Specifically, pathogenic germline mutations in *BRCA2* are associated with higher risk PC at diagnosis and subsequent adverse outcomes.^8^, ^9^

In this study, our objectives were to determine the prevalence of pathogenic germline mutations comprising a panel of penetrant cancer predisposition genes in men with low‐risk PC initially managed with AS and to determine if these mutations were associated with adverse characteristics in men who were treated after initial surveillance or with biopsy reclassification during AS.

## 2 METHODS

### 2.1 Study population

The Canary Prostate Active Surveillance Study (PASS; clinicaltrials.gov NCT00756665) is a multi‐center cohort enrolling men who select AS to manage localized PC and who provide informed consent to use specimens and clinical data for research under institutional review board supervision.^10^, ^11^ In PASS, PSA is measured every 3 months, clinical exams occur every 6 months, and prostate biopsies are performed 6–12, 24, 48, and 72 months post diagnosis.^11^ Specimens, including peripheral blood from which DNA was isolated, were collected at enrollment and study visits every 6 months.

### 2.2 Study design

Since mutations in penetrant cancer predisposition genes such as *BRCA2* are associated with adverse PC outcomes, for our primary analysis we specifically included PASS participants with available blood samples that developed the most adverse characteristics subsequent to study entry (*n* = 170). Adverse characteristics were defined as adverse pathology (AP; ≥GG3, ≥pT3a, or N1) at radical prostatectomy (RP); biochemical recurrence (BCR) after primary therapy (PSA ≥0.2 ng/ml on two measurements after RP, PSA > Nadir+2.0 after radiation, or initiation of salvage treatment in the setting of elevated PSA); or confirmed metastases. To accommodate a prospective cohort with censored failure time outcome, a nested case–control design (NCC) was utilized for the time‐to‐event outcome.^12^, ^13^ All samples with the presence of adverse characteristics by the data extraction date of April 2019 were selected as cases, and two controls were randomly selected from a set of patients who had no evidence of adverse characteristics at time of matching (matched based on time since initial PC diagnosis). The NCC comprises two key features: Feature 1 ‐ it is possible that one sample may serve as a control multiple times based on matching criteria; Feature 2 ‐ it is possible a sample utilized as a control at an earlier timepoint will become a case at a later timepoint due to the development of an adverse characteristic (Figure S1). Based on these features, the number of patients with no evidence of adverse characteristics is less than twice the number of patients with adverse characteristics in the study (*N* = 268 vs. *N* = 169). Secondary endpoints of only BCR and/or metastasis (METS) or of biopsy grade reclassification (upgrading) during AS were also assessed.

### 2.3 Specimen preparation and germline DNAmutation analysis

DNA was isolated from peripheral blood drawn at time of enrollment into Canary PASS utilizing the Qiagen 5’ DNA extraction kits (Qiagen) acchording to the manufacturer's instructions. DNA was quantified by the NanoDrop™ 2000 and 50 ng/μl of DNA in a final volume of 20 μl was plated into BioRad Hard‐shell 96‐well plates. DNA sequencing was performed on all exons of 30 genes associated with cancer predisposition (Table S1) by capture‐based next‐generation sequencing (NGS) by Color Genomics.^14^ Nucleotide variants were classified as “pathogenic,” “likely pathogenic,” or “variant of uncertain significance” (VUS) based on the American College of Medical Genomics classification system,^15^ independently verified (C.C.P.) and sub‐grouped into genes involved in overall DNA repair and DNA double‐strand break (DSB) repair (Table S2). Three samples failed quality control testing (*n* = 2 without adverse characteristics, *n* = 1 with adverse characteristics) and were excluded, resulting in 437 men in the analysis.

### 2.4 Statistical analysis

Descriptive statistics (median, inter‐quartile range (IQR) for continuous variables and count (%) for categorical variables) were utilized to determine baseline characteristics and enumerate mutation calls, both for patients who did and did not develop adverse characteristics, and for those who did and did not undergo adverse reclassification at a surveillance biopsy. The study was powered (80%) to detect three times higher risk of being a case for men carrying a pathogenic mutation, assuming a pathogenic mutation rate among cases of 7%. To evaluate the association of mutations with progression to adverse characteristics in cases and controls selected using an NCC study design, the weighted Cox proportional hazards regression was used.^12^ The study was designed to detect differences in the frequency of pathogenic mutations between patients with and without adverse characteristics as an aggregate of all pathogenic mutations. Further, mutations in individual DNA‐repair genes were interrogated to ascertain differences in the presence of pathogenic mutations between groups. Univariate hazard ratios (HR) were determined for single or grouped gene mutations. Multivariable regression models included log PSA density, which has previously been shown to be associated with AP,^16^ and GG at diagnosis, and race. Secondary analyses were conducted to evaluate the association of mutations with time to development of BCR and/or METS; participants without BCR or METS were censored at date of last study contact. All analyses were conducted using R version 3.5.2 (www.r‐project.org).

## 3 RESULTS

### 3.1 Patient characteristics

**FIGURE 1:**
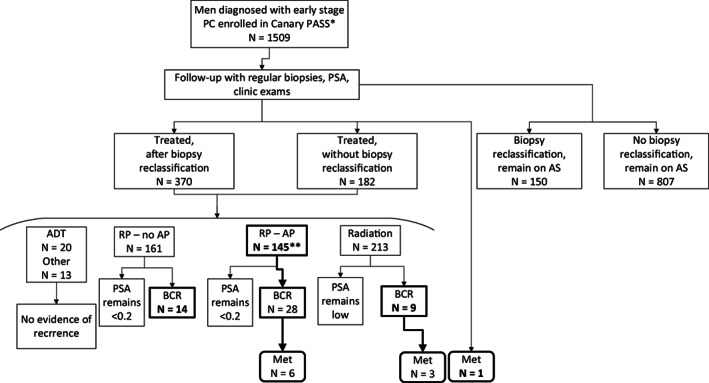
Cancer prostate cancer active surveillance study participant characteristics. cases (in bold font; *N* = 169) in the Canary PASS cohort that developed adverse characteristics after initial active surveillance. Controls (*N* = 268) with no adverse characteristics were matched to cases at the time of the earliest event. RP = radical prostatectomy; AP = adverse pathology; BRC = biochemical recurrence; Met = metastasis. *244 participants with insufficient germline DNA available were excluded prior to selection. **4 AP cases included pN1, 1 which subsequently recurred. The 3 pN1 that did not recur were included in secondary endpoint of recurrence and/or metastasis (*N* = 55)

This study included 169 men who developed an adverse characteristic after initial AS and 268 men with no evidence of adverse characteristics (Figure 1, Table 1 and Table S3). The cases with adverse characteristics included 10 whose cancer metastasized and 42 whose cancer recurred after treatment with no current evidence of metastasis; the remaining 117 had AP at RP with no evidence of recurrence. The 437 participants had a median follow‐up of 7.2 (IQR 5.2–9.3) years and were predominately diagnosed with biopsy Gleason Grade Group (GG) 1 (91%) and clinical stage T1 (87%) cancer (Table 1). The participants were primarily White (91%) with 5% self‐identified as Black and 4% comprising other racial groups. Thirty percent and 32% with and without an adverse characteristic, respectively, had a 1st degree relative with a history of PC.

**TABLE 1:** Characteristics of the study cohort at initial diagnosis

	Adverse characteristic *N* = 169	No adverse characteristic *N* = 268	Total study population *N* = 437
Age, years	63 (57–66)	62 (58–67)	62 (58–66)
Race			
Black	8 (5%)	14 (5%)	22 (5%)
White	152 (90%)	244 (91%)	396 (91%)
Other	9 (5%)	10 (4%)	19 (4%)
Gleason group			
Group 1	149 (88%)	249 (93%)	398 (91%)
Group 2	19 (11%)	16 (6%)	35 (8%)
Group 3	1 (1%)	3 (1%)	4 (1%)
% Positive cores	13.4 (8.3–20.2)	8.3 (8.3–16.7)	10 (8.3–16.7)
PSA, ng/ml	5 (4.2–6.4)	4.8 (3.6–6.5)	4.9 (3.9–6.5)
Prostate size, cm^3^	34.5 (26.7–47.4)	41.1 (30.1–56.2)	39 (28–53.4)
PSA density	0.14 (0.1–0.19)	0.11 (0.08–0.15)	0.12 (0.09–0.17)
Clinical T‐stage			
T1	144 (85%)	235 (88%)	379 (87%)
T2a	25 (15%)	31 (12%)	56 (13%)
T2b	0 (0%)	2 (<1%)	2 (<1%)
BMI	27.3 (24.7–29.8)	27 (24.7–30.2)	27.1 (24.7–30)
1st degree relative with PC	51 (30%)	86 (32%)	137 (31%)
Total follow‐up, years	6.7 (4.4–9)	7.5 (5.5–9.5)	7.2 (5.2–9.3)
# Surveillance biopsy	2 (1–2)	3 (2–3)	2 (1–3)
Grade reclassification	132 (78%)	81 (30%)	213 (49%)
Treatment	168 (99%)	74 (28%)	243 (56%)
Radical prostatectomy	159 (94%)	29 (11%)	187 (43%)
Radiation	9 (5%)	38 (14%)	49 (11%)
ADT	0 (0%)	4 (1%)	4 (1%)
Other	0 (0%)	3 (1%)	3 (1%)

*Note:* Data are summarized as counts (%) for categorical variables and median (IQR) for continuous variables.

Abbreviations: BMI, body mass index, PC, Prostate cancer, Race, other (1 American Indian or Alaska Native, 13 Asian, 1 Native Hawaiian or other Pacific Islander, 1 other, and 3 unknown/prefer not to answer).

### 3.2 Pathogenic and likely pathogenic germline mutations in cancer predisposition genes

Overall, 148 mutations were detected in 22 genes in 123 participants (Table 2). Of these, 32 mutations in 8 genes in 29 of the 437 (6.6%) study participants were classified as pathogenic: *MUTYH* (*n* = 11), *CHEK2* (*n* = 10), *ATM* (*n* = 3), *BAP1* (*n* = 1), *BRCA2* (*n* = 3), *BRCA1* (*n* = 2), *BRIP1* (*n* = 1), and *PMS2* (*n* = 1) (Data S1). The mutations detected in MUTYH, APC, and MITF are common germline variants that are likely to be incidental findings.

**TABLE 2:** Frequency of germline cancer predisposition mutations in men on AS who developed adverse characteristics and those who did not. Pathogenic mutations are listed unless otherwise noted

	Adverse characteristic *N* = 169 N (%)	No adverse characteristic *N* = 268 N (%)	Total study population *N* = 437 N (%)
All mutations incl. Likely and VUS	45 (26.6%)	78 (29.1%)	123 (28.1%)
Pathogenic mutation	11 (6.5%)	18 (6.7%)	29 (6.6%)
DNA damage repair gene	7 (4.1%)	12 (4.5%)	19 (4.3%)
DNA damage repair gene, including VUS	27 (16%)	54 (20.1%)	81 (18.5%)
Double strand break repair gene	7 (4.1%)	11 (4.1%)	18 (4.1%)
BRCA1	0 (0%)	2 (0.7%)	2 (0.5%)
BRCA2	1 (0.6%)	2 (0.7%)	3 (0.7%)
ATM	0 (0%)	3 (1.1%)	3 (0.7%)
CHEK2	5 (3%)	5 (1.9%)	10 (2.3%)
BRCA1/2/ATM	1 (0.6%)	7 (2.6%)	8 (1.8%)

Abbreviations: Incl. likely, Including likely pathogenic mutation; VUS, Variant of uncertain significance.

Pathogenic germline mutations in genes involved in DNA damage repair processes (DRGs), predominantly DSB repair were identified in 19 (4.3%) participants (Table 2). Ten men harbored pathogenic germline mutations in *checkpoint kinase 2* (*CHEK2*), two of which were CHEK2*1100del which is a frequently occurring low penetrance variant found predominantly in individuals of Eastern and Northern European descent,^17^ and four of which were p.I157T another common low penetrance variant.^7^ Pathogenic mutations in *BRCA1* and *BRCA2* were identified in two (0.5%) and three (0.7%) participants, respectively, and three participants harbored pathogenic germline mutations in *Ataxia‐Telangiectasia Mutated* gene (*ATM*) (0.7%). Pathogenic germline mutations in the *BRCA1 associated protein 1* (BAP1) gene and *BRCA1 interacting protein C‐terminal helicase 1* (*BRIP1*) were each found in one participant.

Collectively, there was no association between the prevalence of germline mutations in men with an adverse characteristic versus those without an adverse characteristic when including all mutations or specifically those classified as a pathogenic germline mutation (Table 2). The overall prevalence of pathogenic germline mutations did not differ between men with (5.1%) or without (7.3%) a first degree relative with PC. All of the pathogenic germline mutations were identified in White men, but the low representation of other ancestries precluded meaningful comparisons.

### 3.3 Risk of adverse prostate cancer characteristics in men with DNArepair gene mutations

The frequency of pathogenic germline DRG mutations was similar in men who developed adverse characteristics (*n* = 7 of 169; 4.1%) and those who did not (*n* = 12 of 268; 4.5%) (Table 2). Similar results were observed in the individual genes or when VUS was included (16% in men with an adverse characteristic, 20% of men without an adverse characteristic). Having a pathogenic DRG mutation was not associated with development of adverse characteristics, either in univariate analysis (HR = 0.87, 95% CI: 0.36–2.06, *p* = 0.7), or when adjusted for PSA density, GG at diagnosis, and race (HR = 0.73; 95% CI: 0.29–1.86, *p* = 0.5) (Table 3). Similar results were found for *BRCA1*, *BRCA2*, *ATM* combined, and *CHEK2* individually, and results did not change when VUS was included (Table 3).

**TABLE 3:** Association of pathogenic germline mutations with development of adverse characteristics

	Univariate hazard ratio (95% CI)	Univariate *p*‐value	Multivariate hazard ratio (95% CI)	Multivariate *p*‐value
DNA damage repair gene	0.87 (0.36–2.06)	0.7	0.73 (0.29–1.86)	0.5
Double strand break repair gene	0.94 (0.39–2.26)	0.9	0.81 (0.31–2.08)	0.7
CHEK2	1.43 (0.48–4.28)	0.5	1.48 (0.47–4.67)	0.5
BRCA1/2/ATM	0.23 (0.03–1.8)	0.16	0.18 (0.02–1.44)	0.11

*Note:* Multivariate analysis adjusted for log PSA density, GG at diagnosis, and race.

Abbreviation: CI, confidence interval.

There were 55 cases in which BCR and/or METS developed. Secondary analyses were performed to evaluate the association of mutations with the composite of these endpoints. Pathogenic germline mutations in DRGs were found in 4 of 55 (7.3%) cases with BCR and/or METS, and in 15 of 372 (4.0%) men with no current evidence of BCR or Mets (Table S4). The presence of mutations was not significantly associated with development of recurrence or metastasis (univariate HR = 1.49, 95% CI: 0.5–4.44, *p* = 0.5).

### 3.4 Risk of adverse grade reclassification during active surveillance and germline mutations in cancer predisposition genes

Of the 437 men comprising the study population, 213 (49%) had adverse grade reclassification on a surveillance prostate biopsy. The frequency of a pathogenic germline mutation in the 30 cancer predisposition genes evaluated did not differ substantially between men with or without grade reclassification (5.4% versus 8.0%). The frequency of pathogenic germline mutations in the *BRCA1*, *BRCA2*, and *ATM* gene triad did not differ in men with grade reclassification compared to those without: 4 of 213 (1.9%) versus 4 of 224 (1.8%), respectively, and the results were similar when including VUS in these genes (Table 4). When considering the collective group of genes involved in DNA DSB repair no significant difference in the pathogenic germline mutations prevalence between men with and without grade reclassification was observed: 12 of 213; 5.6% versus 6 of 224; 2.7%, respectively, and this did not differ when including all VUS in these genes (Table 4).

**TABLE 4:** Frequency of germline cancer predisposition mutations in men with and without grade reclassification. Pathogenic mutations are listed unless otherwise noted

	Grade reclassification *N* = 213 N (%)	No grade reclassification *N* = 224 N (%)
All mutations including VUS	65 (30.5%)	58 (25.9%)
Pathogenic mutation	17 (8%)	12 (5.4%)
DNA damage repair gene	13 (6.1%)	6 (2.7%)
DNA DSB repair gene	12 (5.6%)	6 (2.7%)
BRCA1	1 (0.5%)	1 (0.4%)
BRCA2	2 (0.9%)	1 (0.4%)
ATM	1 (0.5%)	2 (0.9%)
CHEK2	7 (3.3%)	3 (1.3%)
BRCA1/2/ATM	4 (1.9%)	4 (1.8%)

Abbreviations: Incl. likely, Including likely pathogenic mutation; VUS, Variant of uncertain significance.

## 4 DISCUSSION

In this study, we sought to determine if inherited mutations in genes associated with cancer predisposition, including a subset involved in DNA repair processes, are associated with the aggressive biological behavior of PC in men on AS ‐ as determined by adverse grade reclassification while on AS, adverse pathology at the time of definitive treatment, or recurrence after treatment. Notably, the frequency of pathogenic mutations in DNA repair genes was low in our cohort: a total of 19 men (4.3%) men harbored a pathogenic mutation in any DNA repair gene, but 10 of these were in *CHEK2*, a gene with unclear associations with aggressive PC.^18^, ^19^

The present cohort included participants with GG2 and GG3 disease, which may suggest the rate of pathogenic germline mutations would be even lower in a cohort comprised of only GG1 disease. Eight men (1.8%) carried a mutation in *BRCA1*, *BRCA2*, or *ATM*, of which only one occurred in the cohort with an adverse characteristic. As there are few prior reports of mutation frequencies in low‐risk patients using AS, we hypothesized that the most aggressive cases in the cohort undergoing AS would harbor a higher frequency of pathogenic mutations, and thus the present study was designed such that if pathogenic mutations were present in 7% of cases and 2% of controls, there would be an 80% power to detect the difference. Our finding of 1.8% of participants harboring a pathogenic mutation in *BRCA1/2* or *ATM* is similar to that of 2.1% recently reported by Carter et al. in their cohort of patients enrolled in AS.^20^ Notably, only 3 men (0.7%) carried a pathogenic germline *BRCA2* mutation of which one had adverse reclassification on biopsy and two did not. These findings are consistent with a prospective study of 18 germline DNA repair gene mutation carriers (*n* = 6 *BRCA2* carriers) diagnosed with PC and selecting AS.^21^ All 18 were classified as GG1 with Gleason 3 + 3 histology and 80% were free from upgrading or radical treatment at a median of 28 months (IQR 8.5–42 months).

Mutations in homologous recombination DRGs have been associated with adverse pathological features at diagnosis such as ductal carcinoma and higher Gleason patterns.^22^, ^23^, ^24^ Thus, an explanation for our low rate of DNA repair mutations could be that individuals with such mutations will generally exhibit histology indicative of higher risk PC and be counseled against AS, and thus comprise a very small proportion of a rigorously selected AS population. Further, though germline mutations in DNA repair‐related genes predispose an individual to cancer, the pathway to neoplasia generally requires loss of the second functional copy of the gene affected. In the setting of a very common malignancy such as PC, cancers in mutation carriers may arise through other oncogenic mechanisms, maintaining a wild‐type copy of the alternate allele, and consequently not manifest an aggressive phenotype that would occur if the cancer developed as a consequence of inactivating the second functional copy of the gene altered in the germline.

Previous reports that examined the association of DNA repair germline mutation and outcomes on AS are conflicting. The aforementioned study from Carter et al reported that germline mutations in *ATM*, *BRCA1*, and *BRCA2* are associated with adverse biopsy grade reclassification in men on AS.^20^ Of 1211 men studied, 26 (2.1%) had a pathogenic mutation in one of these three genes. When considered as a 3‐gene panel, carriers were more likely to have a reclassification event: HR = 1.96 (95% CI 1.004–3.84, *p* = 0.04). Individually neither *ATM* nor *BRCA1* were associated with adverse reclassification, whereas *BRCA2* alone had an adjusted HR of 2.74 (95% CI = 1.26–5.96), indicating that the major contributor to this gene panel was *BRCA2*. Notably, as in our present study, the frequency of germline *BRCA2* mutations was very low at 0.9%, and of the 11 *BRCA2* mutation carriers, 5 did not experience grade reclassification.^20^ Further, Halstuch et al reported that, with relatively limited follow‐up, AS was a feasible strategy for men with low‐risk PC who were carriers of DNA‐repair gene mutations.^21^

There are limitations associated with this study. The lack of information as to the somatic mutation status of the inherited wild‐type allele, or genomic alterations reflecting DNA repair deficiency, limits mechanistic conclusions about the direct role of an inherited mutation driving individual cancer trajectories. The sample size is relatively small relative to the frequency of rare penetrant mutations in the population. The study population has limited diversity with respect to ancestry precluding conclusions generalizing the findings across populations. The use of adverse characteristics or grade reclassification as intermediate endpoints have known misclassification and may not reflect long‐term disease trajectories and outcomes. Central pathology review of the biopsy slides from participants involved in this study is currently underway and may form the premise for future studies to evaluate a correlation between histology patterns and germline aberrations in men on AS.

## 5 CONCLUSIONS

Our study found a very low frequency of pathogenic germline mutations in genes involved in DNA repair in a population of men with a diagnosis of PC meeting criteria for AS. Although this low frequency must be taken into consideration when interpreting the data, no apparent association between germline mutation status and adverse clinical or pathological features was identified. These data do not support routine germline testing for men with a favorable risk of prostate cancer in the absence of other risk factors.

## CONFLICT OF INTEREST

The authors declare no conflicts related to this work. PSN received consulting fees from Janssen, BMS and Pfizer for work unrelated to this study.

## AUTHOR CONTRIBUTIONS

Peter S. Nelson had full access to all the data in the study and takes responsibility for data integrity and data analysis accuracy. Study concept and design: Brady, Nelson, Lin, Newcomb; Acquisition of data: Brady, Nelson, Lin, Newcomb; Analysis and interpretation of data: All authors; Drafting the manuscript: Brady, Nelson; Critical review and revision of the manuscript: All authors; Statistical analysis: Zhu, Zheng; Obtaining funding: Nelson, Lin. Administrative, technical, or material support: All authors; Supervision: Nelson, Lin, Newcomb.

## ETHICS STATEMENT

Patients enrolled in this study provided informed consent to use specimens and clinical data for research under institutional review board supervision. The trial is registered in Clinical Trials.govunder accession: NCT00756665.

## Supporting information


Figure S1
Table S1
Table S2
Table S3
Table S4

Table S5

## Data Availability

The data that supports the findings of this study are available in the supplementary material of this article.
