# Supplementary material for: Germline mutations in penetrant cancer predisposition genes are rare in men with prostate cancer selecting active surveillance
Source: Cancer Med. 2022 Apr 25;11(22):4332–40. doi: 10.1002/cam4.4778 (PMC9678104; doi:10.1002/cam4.4778)
Supplement: Supplementary file 1 — Figure S1 Table S1 Table S2 Table S3 Table S4 [file CAM4-11-4332-s002.docx]

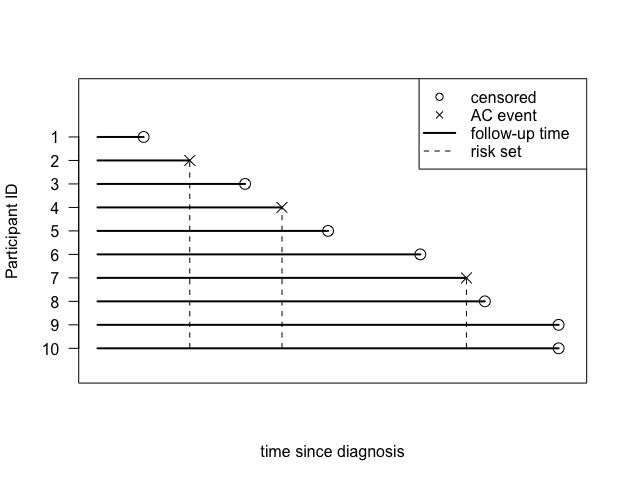


**Supplementary Figure S1:** An illustration of the nested case-control study design.

Figure S1 illustrates the study design with a hypothetical cohort of 10 participants. Each horizontal line represents time from diagnosis to event or censoring time for each participant. We observe the event for Participants 2, 4 and 7. At the event time for Participant 2, Participant 3 to 10 are in the risk set to be randomly selected in a 1:2 ratio. It is possible that Participant 4 is selected as a control for Participant 2 (Feature 1). Participant 5 is in the risk set of Participant 2 and 4’s event time hence it is possible that Participant 5 is selected as a control twice (Feature 2).

**Supplementary Table S1**: Color Genomics’ Hereditary Cancer Panel (30 genes).

| **Gene** |
| --- |
| BRCA1 |
| BRCA2 |
| MLH1 |
| MSH2 |
| MSH6 |
| PMS2 |
| EPCAM |
| APC |
| MUTYH |
| MITF |
| BAP1 |
| CDKN2A |
| CDK4 |
| TP53 |
| PTEN |
| STK11 |
| CDH1 |
| BMPR1A |
| SMAD4 |
| GREM1 |
| POLD1 |
| POLE |
| PALB2 |
| CHEK2 |
| ATM |
| NBN |
| BARD1 |
| BRIP1 |
| RAD51C |
| RAD51D |

**Supplementary Table S2:** Gene sub-groups.

| **Overall DNA repair** | **Double-strand break repair** |
| --- | --- |
| BRCA1  BRCA2  BRIP1  NBN  BARD1  ATM  CHEK2  RAD51C  RAD51D  BAP1  MLH1  MSH2  MSH6  PMS2 | BRCA1  BRCA2  BRIP1  NBN  BARD1  ATM  CHEK2  RAD51C  RAD51D  BAP1 |

**Supplementary Table S3:** Characteristics of men with and without sufficient DNA.

|  | **Men with sufficient DNA**  **N = 1509** | **Men without sufficient DNA**  **N = 244** |
| --- | --- | --- |
| **Age, years** | 63 (58-67) | 63 (58-68) |
| **Race** | | |
| **Black** | 96 (6%) | 21 (9%) |
| **White** | 1341 (89%) | 202 (83%) |
| **Other** | 72 (5%) | 21 (9%) |
| **Gleason Group** | | |
| **Group 1** | 1380 (91%) | 214 (88%) |
| **Group 2** | 122 (8%) | 30 (12%) |
| **Group 3** | 7 (<1%) | 0 (0%) |
| **% positive cores** | 10 (8.3-16.7) | 16 (8.3-23.6) |
| **PSA, ng/mL** | 5 (3.9-6.6) | 5.4 (4.3-7.1) |
| **Prostate Size, cm^3^** | 42.7 (31-57.8) | 43.4 (32.9-60) |
| **PSA Density** | 0.11 (0.08-0.16) | 0.12 (0.09-0.18) |
| **Total follow-up, years** | 6.1 (3.4-8.5) | 1.5 (1-4.1) |
| **Treatment** | 552 (37%) | 46 (19%) |
| **Adverse Characteristic** | 170 (11%) | 7 (3%) |

**Supplementary Table S4:** Frequency of germline mutations in patients with or without evidence of biochemical recurrence and/or metastasis. Pathogenic mutations are listed unless otherwise noted.

|  | **Patients with**  **BCR/mets**  **N = 55**  **N (%)** | **Patients without BCR/mets**  **N = 372**  **N (%)** |
| --- | --- | --- |
| **All mutations including VUS** | 14 (25.5%) | 107 (28.8%) |
| **Pathogenic mutation** | 5 (9.1%) | 24 (6.5%) |
| **DNA damage repair gene** | 4 (7.3%) | 15 (4%) |
| **double strand break repair gene** | 4 (7.3%) | 14 (3.8%) |
| **BRCA1** | 0 (0%) | 2 (0.5%) |
| **BRCA2** | 0 (0%) | 3 (0.8%) |
| **ATM** | 0 (0%) | 3 (0.8%) |
| **CHEK2** | 3 (5.5%) | 7 (1.9%) |
| **BRCA1/2/ATM** | 0 (0%) | 8 (2.2%) |

BCR – Biochemical recurrence, Incl. likely – Including likely pathogenic mutation, Mets – metastasis, VUS – Variant of uncertain significance.
